# Comparison of Three Endodontic Irrigant Regimens against Dual-Species Interkingdom Biofilms: Considerations for Maintaining the Status Quo

**DOI:** 10.3390/antibiotics9090634

**Published:** 2020-09-22

**Authors:** Om Alkhir Alshanta, Saeed Alqahtani, Suror Shaban, Khawlah Albashaireh, William McLean, Gordon Ramage

**Affiliations:** Glasgow Endodontology Group, Glasgow Dental School, School of Medicine, Dentistry and Nursing, College of Medical, Veterinary and Life Sciences, Glasgow G12 8QF, UK; 2151874A@student.gla.ac.uk (O.A.A.); 2195788A@student.gla.ac.uk (S.A.); dr.sarah200988@yahoo.com (S.S.); 2372130A@student.gla.ac.uk (K.A.); William.mclean@glasgow.ac.uk (W.M.)

**Keywords:** Interkingdom, biofilm, *Candida albicans*, *Enterococcus faecalis*, EDTA, NaOCl, HEDP, persistence

## Abstract

Endodontic infections are often interkingdom biofilms, though current clinical management rarely considers this phenomenon. This study aimed to evaluate new and standard endodontic antimicrobial regimens against simple and complex *Candida albicans* and *Enterococcus faecalis* mono- and dual-species biofilms. *C. albicans* and *E. faecalis* mono- and dual-species biofilms were grown upon Thermanox™ coverslips and treated for 5 min with 3% NaOCl, 3% NaOCl followed by 17% EDTA, or 9% HEDP dissolved in 3% NaOCl. The number of cells remaining immediately after treatment at 0 h and after 72 h of regrowth were assessed using real-time quantitative PCR. All three treatment arms showed a similar positive antimicrobial effect on *C. albicans* and *E. faecalis* in both mono- and dual-species biofilms following initial treatment, resulting in ≥98% reduction in colony forming equivalent (CFE). Regardless of species or biofilm type (mono- or dual- species), the antimicrobial effect of NaOCl:HEDP mixture was comparable to that of NaOCl alone, with both showing significant regrowth after 72 h, whereas sequential treatment with NaOCl and EDTA consistently prevented significant regrowth. Our data suggest that sequential irrigation with NaOCl and EDTA remains the antimicrobial strategy of choice as it significantly reduces biofilm persistence and regrowth in our experimental dual-species biofilm conditions.

## 1. Introduction

Apical periodontitis arises from micro-organisms mainly organized as microbial biofilm in the intricate anatomy of the root canal system [1,2]. It is now widely accepted that biofilms rarely exist as single species entities. In fact, they exist as complex, diverse and heterogeneous cellular communities of organisms spanning different phylogenetic kingdoms [3]. Interactions between the contributing organisms can have a negative impact on health through intensification of pathogenicity and increased tolerance to antimicrobial challenge [4]. *Enterococcus faecalis* has been classically associated with endodontic infections, and whilst occasionally detected in primary infections, it is more frequently associated with post-treatment disease [5]. Microbially less well recognised with post-treatment disease, but arguably as important is the yeast *Candida albicans*. Its prevalence in canals of failing root filled teeth varies from one study to another [6,7], though it has been reported to be as high as 36.7% [8]. Interestingly, it is frequently co-isolated from polymicrobial biofilm infections and can interact with a number of different bacteria in a variety of ways [9], particularly driving enhanced antimicrobial tolerance. Given its phenotypic plasticity to change from yeast cells to form long hyphal elements, it creates a physical structure to support and protect bacterial biofilms [10].

The main goal of endodontic treatment is to eliminate or reduce the microbial biofilm burden to a subcritical level that promotes healing of periapical lesions. It is recognised that mechanical debridement and chemical disinfection should be utilised to achieve this aim. A number of chemical disinfection agents have been explored, however, sodium hypochlorite (NaOCl) remains the most widely used irrigant for root canal disinfection. NaOCl is considered the gold standard disinfectant in endodontic applications due to its proven effectiveness against biofilms and its ability to dissolve organic material [11]. It is important to recognise that despite employing robust mechanical and chemical regimens, the complete eradication of microorganisms from the root canal system is difficult to achieve [12]. This is mainly due to the complexity of the root canal system, the ability of microorganisms to invade the dentinal tubules, and the overriding biofilm lifestyle [13]. The complex anatomy of root canal system has been well described by Vertucci [14]. The presence of lateral canals, multiple foramina, deltas, isthmus and c-shaped canals are some forms of the complex anatomy that may render root canal disinfection more challenging. Indeed, recent work from our group has highlighted notable persistence of microorganisms following the use of standard endodontic irrigants (sequential application of NaOCl and EDTA), which has potential clinical relevance when considering polymicrobial biofilms [15]. Recently 1-hydroxyethane 1,1-diphosphonic acid (HEDP) has been suggested as an alternative chelating agent [16]. HEDP is a mild chelating agent that, unlike others can be used simultaneously with NaOCl. This has led to the development of the concept of “continuous chelation” [17], which is in contrast to the sequential chelation that is required when other chelators such as EDTA are utilised. This single chemotherapeutic strategy has the potential to mitigate any tolerance and persistence observed in conventional approaches. Notably, there have been few studies actively addressing the effectiveness of HEDP, and these have been limited to single *E. faecalis* biofilm studies [16,18,19]. In light of the convenience of HEDP as a potential replacement for EDTA, the aim of the present study was to explore the effect of the use of a continuous or sequential chelator on persistence and regrowth of dual-species *C. albicans* and *E. faecalis* biofilms; biofilms representing a tougher and more meaningful challenge.

## 2. Results

### 2.1. Effect of Endodontic Irrigants on Planktonic Cells

Initially, the effect of treatment regimens on planktonic mono-species and dual-species microorganisms was established through planktonic minimum inhibitory concentration (PMIC) tests (Table 1). Data indicated that *C. albicans* was most sensitive to all agents tested compared to *E. faecalis*, except HEDP dissolved in NaOCl. Moreover, apart from this treatment, all dual-species inoculum were equivalent to the MICs of *E. faecalis*. Both organisms were most sensitive to NaOCl alone or along with HEDP. These data provided reassurances that the endodontic irrigants have antimicrobial activity for subsequent experiments, except for HEDP alone (PMIC = 4.5%).

### 2.2. Effect of Endodontic Irrigants on Single Species Biofilms

**Figure 1:**
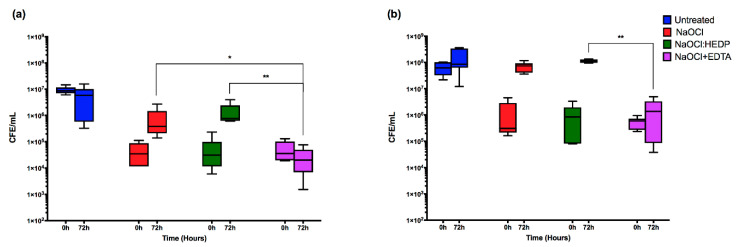
The effect of the three endodontic irrigants on pre-formed 24 h mono-species biofilms. NaOCl alone, NaOCl:HEDP and NaOCl+EDTA were used to treat (**a**) *C. albicans* and (**b**) *E. faecalis*. CFE at 0 h and 72 h are presented as box and whisker plot of each treatment on log_10_ Y axis. Statistical significance between the three treatment conditions at 72 h was presented as * *p* < 0.05, ** *p* < 0.01. Results represent data from three independent experiments.

**Figure 2:**
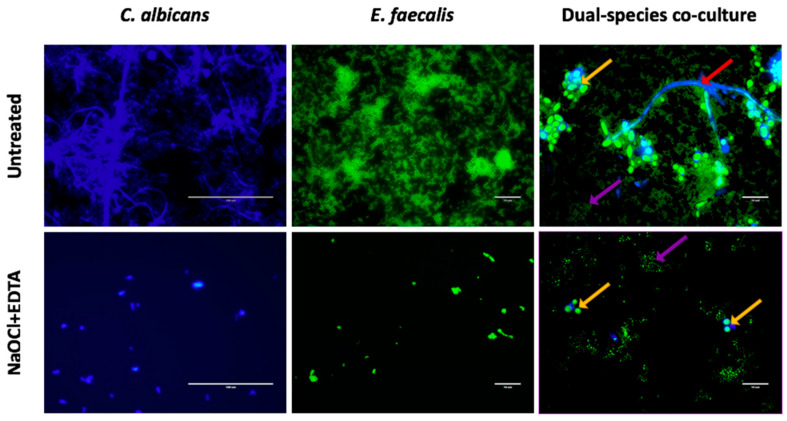
Fluorescence microscopic examination of *C. albicans* mono-species, *E. faecalis* mono-species and dual species biofilms. Upper panel represents untreated biofilms at 72 h. Lower panel represents regrowth of biofilms treated with NaOCl+EDTA at 72 h. *C. albicans* in mono-species and dual-species biofilms was stained blue with calcofluor white while the green fluorescence SYTO 9 stained both *C. albicans* and *E. faecalis* cells. Yellow arrows show *C. albicans* yeast cells, red arrow shows *C. albicans* hyphae and purple arrows show *E. faecalis*. Scale bars is 100 μm in *C. albicans* mono-species and 10 μm in *E. faecalis* mono-species and dual-species co-culture.

Next, we tested each endodontic irrigant against *C. albicans* mono-species biofilms, where it was shown that all the three treatments have a comparably high initial killing effect at 0 h, resulting in >99% reduction in CFE compared to untreated controls (Figure 1a). The median number of CFE/mL in the control group was 8.7 × 10^6^, reducing to approximately ≤3.5 × 10^4^ CFE/mL for all treatment arms (Table 2). After 72 h, NaOCl+EDTA was significantly the most effective at inhibiting subsequent regrowth (*p* < 0.05). Only residual yeast cells can be observed when viewed by Calcofluor staining (Figure 2). Conversely, NaOCl alone and the NaOCl:HEDP mixture exhibited a significant regrowth (*p* < 0.05), showing a 17- and 23.5-fold increase in CFE/mL at 72 h compared to 0 h respectively. There was no significant difference between NaOCl alone and NaOCl:HEDP at both these time points (*p* > 0.05).

For *E. faecalis*, the immediate killing effect of the three treatments at 0 h was comparable to that of *C. albicans*, resulting in ≥98% reduction in CFE compared to untreated controls (Figure 1b). The median number of CFE/mL was reduced from 6.22 × 10^7^ in the control group to ≤8.5 × 10^5^ for all treatments. There was no statistically significant difference between the three treatments at 0 h (Table 2). However, at 72 h *E. faecalis* was more resilient and showed a greater ability to regrow with the NaOCl and NaOCl:HEDP treatments compared to the effect on *C. albicans.* There was approximately a 54- and 66-fold increased cell number at 72 h compared to 0 h with NaOCl alone and NaOCl:HEDP treatments, respectively. Again, NaOCl+EDTA was the most effective in inhibiting regrowth at 72 h, with only a three-fold increase in CFE/mL (*p* < 0.01) (Figure 1b). Small aggregates of cells can be observed retained on the surface of coverslips when viewed by fluorescence microscopy (Figure 2).

### 2.3. Effect of Endodontic Irrigants on Dual Species Biofilms

**Figure 3:**
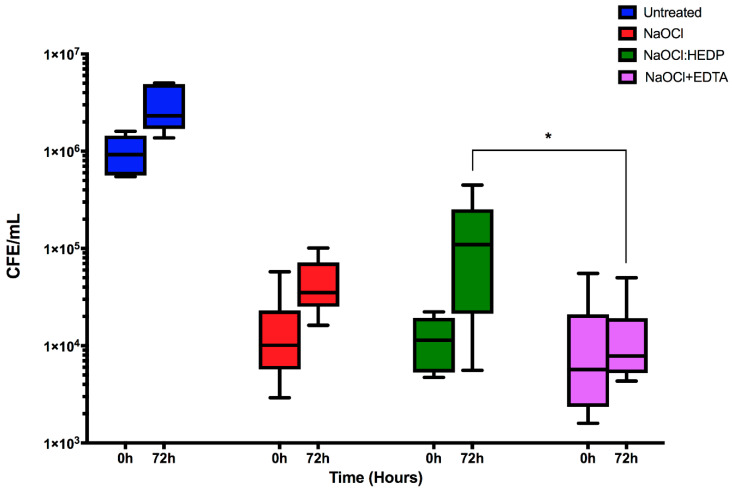
The effect of the three endodontic irrigants on *C. albicans* in pre-formed 24 h dual-species biofilm. NaOCl alone, NaOCl:HEDP and NaOCl+EDTA were used to treat mixed species biofilms and *C. albicans* quantified. Box and whisker plot of CFE at 0 h and 72 h for each treatment. Values were plotted as log_10_ on the Y axis. Statistical significance between the three treatment conditions at 72 h was presented as * *p* < 0.05. Results represent data from three independent experiments.

Next, dual-species biofilms were treated and assessed as described above for mono-species biofilms, and *C. albicans* quantified (Figure 3). The effect of the three treatments at 0 h (immediately after treatment) was similar to that of mono-species biofilms, with all treatments significantly reduced by ≥98.4% compared to controls (9.24 × 10^5^ CFE/mL, *p <* 0.05). NaOCl+EDTA was the most effective regimen overall, but also notably with no significant regrowth detected after 72 h (7.8 × 10^3^ CFE/mL) compared to the initial treatment (5.7 × 10^3^ CFE/mL). Conversely, despite their initial effectiveness both NaOCl alone and NaOCl:HEDP were associated with increased residual biofilm by three- and 12-fold between initial treatment at 0 h compared 72 h post-treatment, respectively. Notably, the residual *C. albicans* cells were significantly lower in NaOCl+EDTA at 72 h compared NaOCl:HEDP treatment (*p* < 0.05, Figure 3).

**Figure 4:**
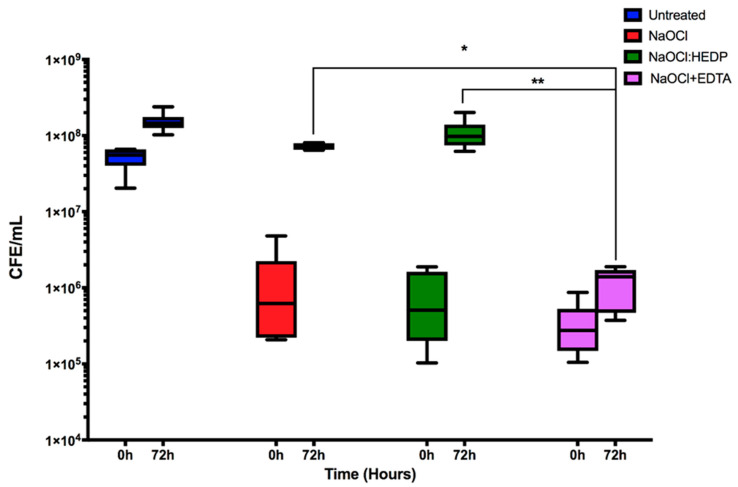
The effect of the three endodontic irrigants on *E. faecalis* in pre-formed 24 h dual-species biofilm. NaOCl alone, NaOCl:HEDP and NaOCl+EDTA were used to treat mixed species biofilms and *E. faecalis* quantified. Box and whisker plot of CFE at 0 h and 72 h for each treatment. Values were plotted as log10 on the Y axis. Statistical significance between the three treatment conditions at 72 h was presented as * *p* < 0.05, ** *p* < 0.01. Results represent data from three independent experiments.

Finally, dual-species biofilms were treated and assessed as described above for mono-species biofilms, and *E. faecalis* quantified. In dual-species, *E. faecalis* was significantly reduced immediately after all three treatments by ≥97.4% (*p* < 0.01), though neither treatment was more effective than one another (Figure 4). However, both NaOCl and NaOCl:HEDP treatments showed significant levels of regrowth after 72 h (*p* < 0.01), returning to baseline control levels of up to and greater than 7 × 10^7^ CFE/mL (55- and 137-fold increase, respectively (Table 2)). Conversely, for the NaOCl+EDTA treatment after 72 h only a five-fold increase was observed, which was significantly reduced compared to NaOCl (*p* > 0.05) and NaOCl:HEDP (*p* < 0.01). This is visually evident when microscopic examination is performed (Figure 2). Here we show that compared to the untreated dual-species biofilm where coccus, yeasts and hyphae are observed, that when treated and observed at 72 h then very low levels of representative morphotypes are evident.

## 3. Discussion

In the present study we tested the anti-biofilm properties of HEDP, which in endodontics is used as a mild chelating agent to remove the smear layer. In contrast to other chelating agents such as EDTA, it can be mixed with NaOCl to simplify chemical cleaning. It can be also dissolved in normal saline and used separately to remove the smear layer. Its effectiveness in smear layer removal and its effect on radicular dentine has been previously evaluated and shown to be as effective as NaOCl followed by 17% EDTA, but resulted in less dentine erosion and is less toxic [20]. However, augmentative antimicrobial properties of chelating agents would also be highly desirable in root canal disinfection. Here, its antimicrobial properties were compared with standard endodontic irrigants such as NaOCl alone, and more importantly those of NaOCl followed by EDTA. This later sequential chelation system is considered the gold standard in irrigation protocols as it allows for not only biofilm disruption but also smear layer removal. Results of the present study show that HEDP has no significant impact on the antimicrobial activity of NaOCl, either positively or negatively, within a simple mono-species and more complex dual-species challenge. Indeed, our studies have indicated that treatment with NaOCl followed by EDTA is consistently more effective, though caveats exist with respect to resilient biofilm populations.

Our direct post-treatment data (0 h) for both mono- and dual-species *E. faecalis* biofilms did not show a significant difference between either of the treatment arms. In contrast, when considering regrowth after treatment over time, NaOCl+EDTA was significantly more effective in inhibiting regrowth. HEDP alone (dissolved in normal saline) has no antimicrobial effect under the experimental conditions of the present study. Contrary to this, comparison of the antimicrobial effect of combined NaOCl and dual rinse HEDP to a standard NaOCl+EDTA irrigating sequence against *E. faecalis* grown inside root canals from extracted human teeth showed that a NaOCl:HEDP mixture resulted in a significantly lower residual bacterial viability [21]. However, Zehnder, Schmidlin [16] found that HEDP did not affect *E. faecalis* viability and did not impact the antibacterial activity of NaOCl, findings echoed elsewhere [18,19]. These experimental conclusions are likely explained by differences in the in vitro substrates used for these studies, so some caution must be exercised in making bold clinical judgements based on these findings.

Irrespective of treatment regimen tested, there were still remaining populations of *C. albicans* and *E. faecalis* following initial treatment which were able to persist and regrow. The antifungal effect of the traditional chelator EDTA is well-documented in the literature [22,23], and as discussed in our previous work EDTA significantly reduced *C. albicans* regrowth [15]. Nevertheless, it has limited or no antibacterial activity on *E. faecalis* at any time or concentration [24,25]. Therefore, it may be somewhat unexpected to see that EDTA significantly inhibited *E. faecalis* regrowth after NaOCl treatment. It is possible that this observed effect of EDTA is related to its membrane permeabilising effect. Lipopolysaccharide molecules have a strong affinity for divalent cations which are required for the thermodynamic stability of the cell membrane [26]. EDTA disrupts the outer membrane of gram-negative bacteria by removing divalent cations [27]. This membrane destabilising effect may be potentiated by the prior treatment of the bacterial cells with NaOCl. HEDP in our experiments did not show an effect, this may be a result of its weaker chelating activity.

Perhaps the most well studied mechanism of Candida-bacteria interactions is physical attachment. A scaffold of hyphae within a biofilm provides a potential niche for the colonization of various gram-positive and gram-negative bacteria, a phenomenon that has been termed as a “mycofilm” [28]. Increasing evidence suggests that *E. faecalis* and *C. albicans* are usually found co-isolated in persistent endodontic infections [29]. Understanding the inter-kingdom interaction between bacteria and fungi in endodontic infections might help in developing more effective treatment strategies. We have previously demonstrated that *C. albicans* is resilient with high regrowth potential following NaOCl treatment [15]. These findings were confirmed in the present study for mono-species biofilms. However, when co-cultured with *E. faecalis*, *C. albicans* become more susceptible to treatment with decreased ability for regrowth in comparison to their mono-species biofilms. This can be explained due to the interaction between the two species. *E. faecalis* has been shown to inhibit hyphal morphogenesis and biofilm formation in *C. albicans* [30]. Cruz and colleagues also showed that *C. albicans* and *E. faecalis* attenuate each other’s virulence in a *Caenorhabditis elegans* model. This might also explain the low fungal prevalence in endodontic infections, although this effect may be also modified by the conditions of the environment in which this interaction occurred. *C. albicans* virulence attenuation was largely due to the inhibition of hyphal morphogenesis, a major virulence factor in *C. albicans*. However, we did not find that *C. albicans* affected *E. faecalis* response to irrigants in dual-species biofilms. Ishijima, Hayama [31] also showed that heat killed *E. faecalis* EF2001 [commercially available probiotic) administered orally has a protective effect against oral candidiasis of mice tongue. Although the effect of *C. albicans* on *E. faecalis* is partially revealed, it still unknown how *C. albicans* affects *E. faecalis* virulence and response to treatment. Future work should explore the molecular basis for the bidirectional modulation of *E. faecalis* and *C. albicans* behaviour and how that would affect these microorganism’s’ response to endodontic treatment.

As outlined previously, the yeast *C. albicans* and bacteria *E. faecalis* have been frequently isolated from secondary endodontic infection. Historically, both have been used as model endodontic pathogens to test various endodontic antimicrobials. It is clear from the findings of the present study that the use of mono-species models is no longer appropriate. Any model system must recognise the potential for inter-species/inter-kingdom interactions. The impact of *E. faecalis* on *C. albicans* ability to persist and regrow is both interesting and important, as these have a significant impact on how we may view the effectiveness of tested irrigants or medicaments. As such, further studies are required to explore and validate model systems of endodontic disease. This study also highlights the importance of persistence and regrowth in endodontic disease, albeit in a limited number of clinical strains. The present model represents an open system with no anatomical irregularities or complex topography for biofilm to persist, yet we still demonstrate persistence. This is significant problem and further work is required to optimise irrigation strategies and other adjuncts to achieve adequate disinfection.

These results indicate the need for additional work to elucidate the potential clinical implications should a continuous chelation strategy be adopted. *C. albicans* is significantly more susceptible to NaOCl and NaOCl:HEDP treatment when co-cultured with *E. faecalis*, suggesting some level of interkingdom antagonism. Future work should investigate the effect of different endodontic antimicrobials against multi-species biofilms on dentine substrate to allow for wider interspecies interactions to be assessed.

## 4. Materials and Methods

### 4.1. Microbial Growth Conditions and Standardisation

*C. albicans*, a clinical isolate from oral rinse of patients attending restorative clinic at Glasgow Dental Hospital and School for routine dental care was used [32]. This isolate has been previously characterised in terms of antifungal susceptibility, proteolytic activity and biofilm formation ability [15,33,34]. The strain was maintained on Sabouraud’s dextrose agar (SAB (Sigma–Aldrich, Dorset, UK)) plates at 30 °C for 48 h. *E. faecalis* root canal clinical isolate ER5/1 was used [35]. The isolate was cultured on 5% horse blood Columbia agar plate and incubated in 5% CO_2_ at 37 °C for 24 h. An overnight culture was then prepared in yeast peptone dextrose ((YPD) (Sigma-Aldrich, Dorset, UK)) for *C. albicans* and in Brain Heart Infusion ((BHI) Sigma–Aldrich, Dorset, UK) for *E. faecalis. C. albicans* culture was incubated at 30 °C at 120 rpm in an orbital shaker (IKA KS 4000 i control, Berlin, Germany) for 18 h while *E. faecalis* culture was incubated in 5% CO_2_ at 37 °C for 18 h. Cells in cultures were then pelleted by centrifugation for 5 min at 3000 rpm and washed twice with PBS. Finally, yeast cells were counted using a haemocytometer and bacterial cells were standardized to OD_600_ of 0.3 which equivalent of 2 × 10^8^ cells/mL using colorimeter.

### 4.2. Planktonic Minimum Inhibitory Concentration

First, to ensure the active agents were effective against planktonic (free-floating cells), we determined the PMIC for mono-species and dual-species microorganisms. 3% NaOCl (Parcan, Septodont, Saint-Maur-des-Fosses, France), 17% EDTA (Endo-Solution, CERKAMED, Stalowa Wola, Poland) and 9% HEDP [(Dual Rinse HEDP*^®^* Medcem GmbH, Weinfelden, Switzerland) dissolved in either 3% NaOCl or normal saline as per manufacturer’s instructions] were tested using broth microdilution method according to the M27-A3 standard for fungi [36] and the M07-A10 standard for *E. faecalis* [37]. Briefly, *C. albicans* was adjusted to a cellular density of (2 × 10^4^ cells/mL) into 1:1 *v*/*v* mixture of Roswell Park Memorial Institute (RPMI)-1640 (Sigma–Aldrich, Dorset, UK)/Todd Hewitt broth (THB) (Sigma–Aldrich, Dorset, UK) growth media. *E. faecalis* cells were adjusted to a density of (2 × 10^5^ cells/mL). Serial double-fold dilutions of each treatment were performed in a 96-well round-bottom microtiter plates (Corning Incorporated, Corning, NY, USA) using 1:1 *v/v* RPMI/THB media. Treatment concentrations were ranging from (1.5–0.0029%) for NaOCl, (8.5–0.066%) for EDTA and (4.5–0.035%) for HEDP dissolved in 3% NaOCl or normal saline. After serial dilution of treatments, 100 μL of standardized microorganisms were added to 100 μL of each concentration and plates were incubated in 5% CO_2_ at 37 °C for 24 h. All PMIC tests contained 4 technical repeats and performed at 3 different occasions. Finally, the PMIC concentration was determined as the lowest concentration of each treatment that prevents visible growth or colony formation at the bottom of the plate wells.

### 4.3. Biofilm Development, Treatment and Regrowth Assessment

Next, treatments were tested against *C. albicans* and *E. faecalis* mono-species biofilms (surface attached cells). A cellular density of (1 × 10^6^ cells/mL) for *C. albicans*, (1 × 10^7^ cells/mL) for *E. faecalis* was prepared in 1:1 *v*/*v* RPMI/THB growth media. 24 h biofilms of mono-species and dual-species were then formed onto sterile Thermanox™ Coverslips (Thermo Fisher Scientific, Paisley, UK) contained in a polystyrene, 24-well microtiter plates (Corning Incorporated, Corning, NY, USA) and incubated in 5% CO_2_ at 37 °C. Afterwards, unattached cells were removed by washing with PBS and biofilms treated for 5 min by adding 500 μL of either 3% NaOCl only, 3% NaOCl followed by 17% EDTA (5 min each) or 9% HEDP dissolved in 3% NaOCl. Untreated controls of each biofilm were also included. After the 5 min treatment, 500 μL of 5% sodium thiosulfate (Fisher Chemicals, London, UK) was used for 10 min at room temperature to inactivate NaOCl and NaOCl:HEDP mixture, while 500 μL of Dey Engley Neutralising broth (Sigma-Aldrich, London, UK) was used for 15 min at 37 °C to inactivate EDTA. Treatments and neutralizers were removed, and coverslips were washed with PBS. Neutralizers were also added to untreated controls to ensure that the observed effect is solely due to applied treatments and not neutralizers. To further assess the effect of treatments on biofilms’ ability of to persist and regrow, 500 μL of a fresh 1:1 *v*/*v* RPMI/THB growth media was added to the treated and washed biofilms and plates re-incubated for further 72 h in 5% CO_2_ at 37 °C. As an outcome measures, the number of cells remaining immediately after treatment (0 h) and those regrowing after 72 h of reincubation with growth media (72 h) was assessed using real-time quantitative polymerization chain reaction (qPCR). All treatment experiments were performed in duplicates and repeated at 3 different occasions.

### 4.4. Quantitative Analysis Using Real-Time Quantitative PCR

Thermanox™ coverslips were first washed with PBS and sonicated in 1 mL of PBS at 35 kHz in an ultrasonic water-bath (Fisher Scientific, Paisley, UK) for 10 min to dislodge cells. DNA was extracted from sonicated samples using the QIAamp DNA mini kit, as per manufacturer’s instructions (Qiagen, Crawley, UK). PCR was carried out as previously prescribed [38], using the Step-One plus real time PCR machine and StepOne software V2.3 (Life Technologies, Paisley, UK). Briefly, *C. albicans*/E*. faecalis* DNA was added to a PCR mastermix containing Fast SYBR^®^ Green (Thermo Fisher Scientific, Paisley, UK) and specific forward and reverse primers (Table 3) within irradiated RNase-free water. Serial dilutions of bacterial/fungal DNA extracted from 1 × 10^8^ cells/mL were also included to create a standard curve for each species. The applied thermal cycles were as follow: 50 °C for 2 min, 95 °C for 2 min, 40 cycles of 95 °C for 3 s and 60 °C for 30 s. Finally, colony forming equivalents (CFE) was calculated in relation to each species standard curve.

### 4.5. Fluorescence Microscopic Imaging

Thermanox™ coverslips from each treatment condition in the two time points (0 h and 72 h) were washed with PBS. 5 μM calcofluor white (Invitrogen, Paisley, UK) which specifically stains chitin and beta-glucans of fungal cell wall was used to stain *C. albicans* while 5 μM SYTO9 (Thermo Fisher Scientific, Paisley, UK) was used to stain both bacterial and fungal cells with high affinity to nucleic acids. After incubating the samples in the dark for 20 min, excess stain was washed with sterile water. Afterwards, stained samples were fixed with 2% paraformaldehyde for 1 h and imaged using EVOS FL Cell Imaging system (Thermo Fisher Scientific, Waltham, MA, USA).

### 4.6. Statistical Analysis

GraphPad Prism (version 7.0 d) was used for graphs production, data distribution and statistical analysis. D’Agostino-Pearson omnibus normality test was used to test the normal distribution of the data. For statistical analysis, non-parametric Kruskal–Wallis test with Dunn’s multiple comparison test was used to compare CFE values of the three treatment conditions at 0 h and 72 h. Mann–Whitney test was also used to compare mono- and dual-species biofilms of the same treatment.

## 5. Conclusions

Although continuous chelation is an admirable goal, simplifying protocols and potentially minimising the risk of deleterious interactions between irrigants, the current work raises questions of whether we should depart from sequential irrigation. The use of NaOCl followed by EDTA has an enhanced effect on biofilm disruption and significantly reduces *C. albicans* and *E. faecalis* persistence and regrowth. The current study also highlights the need for appropriate biofilm model choices. Both inhibitory and promotory effects can be observed between the participating microorganisms within the biofilm, evidenced here where *C. albicans* is significantly more susceptible to NaOCl and NaOCl:HEDP treatment when co-cultured with *E. faecalis*. It may be that with the addition of further complexity within the biofilm model that the interactions between microorganisms modifies to an even greater extent irrigation effectiveness.

## Figures and Tables

**Table 1:** Susceptibility of planktonic *Candida albicans*, *Enterococcus faecalis* and their co-cultures to sodium hypochlorite, EDTA and HEDP. Numbers represent PMIC values.

Endodontic Irrigant	*C. albicans*	*E. faecalis*	Co-Culture
**NaOCl**	0.093%	0.187%	0.187%
**EDTA**	0.13%	0.265%	0.265%
**HEDP: NaOCl**	0.093%	0.093%	0.187%
**HEDP: saline**	2.25%	4.5%	4.5%

**Table 2:** The median number and range of CFE/mL for untreated controls, NaOCl, NaOCl:HEDP and NaOCl+EDTA treatment at 0 h and 72 h.

Biofilm Type	Time Point	Treatment	Median CFE/mL	Range (Minimum to Maximum)
***C. albicans* mono-species**	0 h	Untreated control	8.70 × 10^6^	(6.05 × 10^6^ to 1.46 × 10^7^)
		NaOCl	3.47 × 10^4^	(1.16 × 10^4^ to 1.13 × 10^5^)
		NaOCl:HEDP	3.10 × 10^4^	(5.97 × 10^3^ to 2.34 × 10^5^)
		NaOCl+EDTA	3.57 × 10^4^	(1.89 × 10^4^ to 1.32 × 10^5^)
	72 h	Untreated control	5.83 × 10^6^	(3.26 × 10^5^ to 1.58 × 10^7^)
		NaOCl	3.82 × 10^5^	(1.40 × 10^5^ to 2.71 × 10^6^)
		NaOCl:HEDP	7.76 × 10^5^	(6.07 × 10^5^ to 3.99 × 10^6^)
		NaOCl+EDTA	2.01 × 10^4^	(1.53 × 10^3^ to 7.65 × 10^4^)
***E. faecalis* Mono-Species**	0 h	Untreated control	6.22 × 10^7^	(2.19 × 10^7^ to 1.04 × 10^8^)
		NaOCl	3.11 × 10^5^	(1.64 × 10^5^ to 4.53 × 10^6^)
		NaOCl:HEDP	8.51 × 10^5^	(8.01 × 10^4^ to 3.36 × 10^6^)
		NaOCl+EDTA	5.97 × 10^5^	(2.37 × 10^5^ to 9.57 × 10^5^)
	72 h	Untreated control	8.61 × 10^7^	(1.22 × 10^7^ to 3.64 × 10^8^)
		NaOCl	7.41 × 10^7^	(3.62 × 10^7^ to 1.18 × 10^8^)
		NaOCl:HEDP	1.10 × 10^8^	(9.44 × 10^7^ to 1.36 × 10^8^)
		NaOCl+EDTA	1.38 × 10^6^	(3.81 × 10^4^ to 4.989 × 10^6^)
***C. albicans* Dual-Species**	0 h	Untreated control	9.24 × 10^5^	(5.472 × 10^5^ to 1.60 × 10^6^)
		NaOCl	1.01 × 10^4^	(2.92 × 10^3^ to 5.77 × 10^4^)
		NaOCl:HEDP	1.14 × 10^4^	(4.724 × 10^3^ to 2.23 × 10^4^)
		NaOCl+EDTA	5.70 × 10^3^	(1.60 × 10^3^ to 5.54 × 10^4^)
	72 h	Untreated control	2.31 × 10^6^	(1.37 × 10^6^ to 5.01 × 10^6^)
		NaOCl	3.52 × 10^4^	(1.62 × 10^4^ to 1.01 × 10^5^)
		NaOCl:HEDP	1.10 × 10^5^	(5.59 × 10^3^ to 4.49 × 10^5^)
		NaOCl+EDTA	7.85 × 10^3^	(4.34 × 10^3^ to 4.50 × 10^4^)
***E. faecalis* Dual-Species**	0 h	Untreated control	5.54 × 10^7^	(2.935 × 10^7^ to 6.61 × 10^7^)
		NaOCl	6.22 × 10^5^	(2.081 × 10^5^ to 4.81 × 10^6^)
		NaOCl:HEDP	5.10 × 10^5^	(1.029 × 10^5^ to 1.89 × 10^6^)
		NaOCl+EDTA	2.76 × 10^5^	(1.05 × 10^5^ to 8.70 × 10^5^)
	72 h	Untreated control	1.46 × 10^8^	(1.03 × 10^8^ to 2.39 × 10^8^)
		NaOCl	7.24 × 10^7^	(6.44 × 10^7^ to 8.06 × 10^7^)
		NaOCl:HEDP	9.76 × 10^7^	(6.21 × 10^7^ to 2.02 × 10^8^)
		NaOCl+EDTA	1.39 × 10^6^	(3.73 × 10^5^ to 1.89 × 10^6^)

**Table 3:** *C. albicans* and *E. faecalis* primers for qPCR.

Primer	Sequence (5′—3′)	References
***C. albicans***	ITS3—GCA TCG ATG AAG AAC GCA GC ITS4—TCC TCC GCT TAT TGA TAT GC	[39]
***E. faecalis***	F—CAAACTG TTGGCATTCCACAA R—TGGATTTCCTTTCCAGTC ACTTC3	[40]
